# A Novel Approach to Calculate the Spatial–Temporal Correlation for Traffic Flow Based on the Structure of Urban Road Networks and Traffic Dynamic Theory

**DOI:** 10.3390/s21144725

**Published:** 2021-07-10

**Authors:** Mao Du, Lin Yang, Jiayu Tu

**Affiliations:** School of Mechanical Engineering, Shanghai Jiao Tong University, Shanghai 200240, China; dumaosjtu@sjtu.edu.cn (M.D.); tujiayu@sjtu.edu.cn (J.T.)

**Keywords:** dynamic programming, intelligent transportation systems, operation characteristics, spatial–temporal correlation, urban road network

## Abstract

Determining the spatial–temporal correlation (STC) between roads can help clarify the operation characteristics of road traffic. Moreover, this correlation affects the utilization quality of traffic data in related research fields. Therefore, it is of significance to provide more reasonable correlation information for other research, such as in traffic speed prediction. Most of the traditional correlation calculation methods for traffic are based on only statistical theory. These methods are simple, but their ability to explain the actual phenomenon is limited due to the lack of consideration of the actual traffic operation characteristics. Therefore, to provide more reasonable correlation information between roads, this paper analysed the influence mechanism of urban traffic based on the traffic dynamic model, and two parameters, traffic complete influence time and traffic correlation strength, were proposed to bring physical meaning to the calculation of STC. Then, an improved calculation model of the STC between different roads considering the adjacency between roads was proposed in this paper. Finally, this paper verified this method against two common traditional methods through different experiments. The verification results show that the calculation method proposed in this paper has better interpretability for the STC between different roads and can better reveal the internal traffic operation characteristics of the road network.

## 1. Introduction

With the continuous development of technology, intelligent transportation systems (ITSs) have emerged as an effective means to alleviate congestion [1]. The use of ITSs can help enhance the operational efficiency of traffic and energy efficiencies of vehicles [2]. The correlation of road traffic mainly reflects the degree of interaction between different roads in the past period, which is of great significance for the future development of traffic state and traffic control research [3,4,5]. Owing to the time-varying characteristics of traffic flow and the spatial distribution characteristics of the road structure, urban traffic data exhibit a notable spatial–temporal characteristic.

The establishment of a scientific and reasonable calculation method for the spatial–temporal correlation (STC) is critical to realize urban traffic research, for example, for the identification of key roads, analysis of the road network topology and development of short-term traffic prediction algorithms [6,7,8].

However, after the investigation, it can be found that the current research did not provide in-depth research on the correlation between traffic states. Most studies in the field of transportation use statistical principles to measure the correlation between roads.

In fact, in the field of transportation, most of the calculation methods only calculate the similarity of two traffic series data but cannot truly judge whether there is traffic correlation between them. For example, a certain road is congested at 8:00 a.m., while its adjacent road is congested at 8:00 p.m. According to the principle of statistics, the two traffic states are very similar, but there is obviously no correlation between them. Alternatively, two roads with a distance of 20 km are congested in the same period of time, but in fact, the traffic states between them do not affect each other, so there is also no correlation between them. Thus, studies based on only the principle of statistics cannot truly reveal the internal correlation characteristics of traffic states, and the correlation results may be consistent with the actual situation.

The traffic network of human society has very complex characteristics, and it is impossible to model it completely. However, with the continuous development of science and theory, various traffic flow theories and modelling methods have been proposed [9]. These models and methods bring more physical meaning to the related research in the field of transportation, and they are helpful to explore the origin and evolution of urban traffic flow.

The major shortcomings of the current research on the correlation of traffic flow are the lack of analysis on the interaction between traffic statuses and the lack of physical support and interpretation of the calculation results. Therefore, in view of the prominent role of traffic models in revealing the dynamic changes in traffic flow, this paper considers the introduction of a traffic dynamic model to improve the calculation method of traffic flow correlation.

In view of the different levels of research and application, these methods can be divided into three categories according to the space metric of the road networks: microscopic, mesoscopic and macroscopic traffic flow analysis. Microscopic traffic flow analysis focuses on the traffic flow characteristics on a point or an end face, including the car-following model [10], lane changing model [11], cellular automaton model [12], etc. Mesoscopic traffic flow analysis studies the traffic flow characteristics of road sections. for example, headway distribution model [13], queue model [14], and so on.

Different from microscopic and mesoscopic traffic flow analysis, macroscopic traffic flow analysis (MTFA) pays attention to the traffic flow characteristics of areas of road networks, such as the average speed and traffic flow over a period of time [15]. Compared with the former two methods, MTFA has the advantages of fewer parameters and simple calculation. It can effectively describe the operation characteristics of traffic flow [16] and has the ability to explore the overall regional traffic operation law [17]. This is of great significance to future traffic state prediction and traffic flow control of urban road networks [18,19]. Among them, the LWR model introduces the theoretical knowledge of fluid mechanics into the study of traffic flow, which makes the study of macrotraffic more physically meaningful, so it has been widely used in the field of traffic research [20]. AliA et al. used macroscopic fundamental diagrams (MFDs) to investigate perimeter traffic flow control for traffic congestion control in heterogeneously congested cities. A robust control of the two-region MFD system is presented to achieve good performance in perimeter traffic flow control [21].

In addition, the operation of traffic flow on urban road networks is closely related to the structural characteristics of road networks. It is undeniable that the urban road network has the characteristics of multiple branches and lanes. The traffic state of the same road will be affected by the traffic state of multiple roads at the same time. It is worth noting that there is no direct impact between the non-directly adjacent roads, and the traffic transfer between them is mainly realized through the transfer path between the roads. Therefore, the correlation between indirect adjacent roads is bound to be affected by the correlation between two adjacent roads on the whole transmission path.

Therefore, considering that the macro traffic model has the advantages of simple calculation and can reveal the dynamic changes of traffic flow, combined with the structural characteristics of urban road networks, this paper proposes an STC calculation method based on a traffic dynamic model for the first time. Experiments are conducted to verify the proposed method systematically.

Three primary contributions of this paper are as follows:

First, based on the traffic dynamic model (LWR model), this paper explores the influence characteristics of urban traffic flow. The parameter of traffic complete influence time is proposed to measure the influence time between different roads for the first time.

Second, considering the multibranch characteristics of the urban road network structure, combined with the separation and confluence mechanism of intersections, the traffic correlation strength is proposed to effectively characterize the influence strength of different roads on the same road.

Third, based on these two parameters, in view of the different influence mechanisms of adjacent roads and non-adjacent roads, this paper creatively put forward the idea of establishing the corresponding STC calculation method according to whether the roads are directly adjacent. To integrate the structural characteristics of urban roads into the calculation process of STC, the calculation results are more explanatory.

The proposed method was verified through different experiments. Compared with the calculation method based on statistical principles, the calculation method proposed in this paper has better interpretability for the STC between different roads and can better reflect the internal traffic operation characteristics of the road network. In addition, the results calculated by the proposed method in this paper show that the traffic correlation decreases with increasing time delay between traffic states and increases with increasing road importance and traffic circulation frequency. Therefore, it indirectly verifies the rationality of this method.

The remainder of this paper is organized as follows. In Section 2, the current research progress of STC is discussed. In Section 3, combined with the characteristics of traffic flow and urban road network structure, this paper proposes two physical parameters to improve the measurement of STC. In Section 4, the corresponding STC calculation method is constructed according to whether the roads are directly adjacent. Section 5 compares the proposed method in this paper with two statistical-based methods. Finally, Section 6 concludes the paper.

## 2. Related Work

The time-varying characteristics of traffic flow and the spatial distribution characteristics of roads cause these traffic data to have obvious spatial–temporal characteristics. Therefore, in the development of a prediction algorithm that is based on multi-sensors, an increasing number of scholars also consider the spatial–temporal correlation (STC) characteristics of traffic in the prediction algorithm [22]. A scientific and reasonable correlation of urban traffic networks is very important for urban traffic research, identification of key roads, analysis of road network topology and short-term traffic prediction algorithms [23].

Based on complex networks, Tang, J. and Wang, Y. analyses the time characteristics of traffic data generated by urban road networks [24]. Complex networks are constructed by using the correlation coefficient among days to measure the complexity of traffic time series at different temporal scales.

Yang and Gar-on investigated the spatiotemporal dependency of traffic flow using cross-correlation analysis and then discusses its implications in terms of traffic forecast ability and real-time data effectiveness [25]. Duan et al. [26] proposed a unified spatial–temporal model for short-term road traffic prediction. This model incorporated the physical factors that potentially affect the variation in the STC into a series of parameters. Another study [27] investigated the inclusion of the STC and interaction into a multivariate random-parameter Tobit model with different severity outcomes. The estimation results show that spatial and temporal effects and their interactive effects are significant and that the spatial and interactive effects have strong correlations across injury severities. A Bayesian spatiotemporal model was proposed to measure the association between the crash frequency and possible risk factors [28]. The proposed model can also accommodate the unstructured random effect, and spatio-temporal correlation and interactions. Guo et al. [29] utilized the weighted degree and impact distance as two major measures to identify the most influential locations. A road segment with a larger weighted degree or larger impact distance suggests that its traffic flow can strongly influence neighboring road sections driven by the congestion propagation.

In regard to the STC calculation of the traffic state, the following correlation calculation methods are often used for analysis:

(1) Time autocorrelation coefficient. This statistical index is used to analyse the correlation degree between the states of the same road at any two instances. However, this index describes only the correlation degree of time and disregards the spatial properties [30].
(2) Spatial autocorrelation coefficient. This coefficient reflects the correlation degree of the attribute variables with a certain regularity in different spatial positions. The common statistical indexes for the spatial autocorrelation analysis are Moran’s I index and Geary’s C index [31].
(3) STC. The change characteristics of the traffic information considering both the time and space dimensions are analysed. To date, many scholars have extended several indexes to measure the STC, such as the simple spatial–temporal autocorrelation index (ST-ACF) and cross-correlation function (CCF) [32,33,34]. The common calculation methods of STC include:
  (a) ST-ACF
    (1) $$\begin{array}{l} \rho_{lk}\left(s\right)=\frac{\sum_{i=1}^{N}\sum_{t=1}^{T}L^{l}x_{i}\left(t\right)L^{k}x_{i}\left(t+s\right)}{\sqrt{\sum_{i=1}^{N}\sum_{t=1}^{T}{\left(L^{l}x_{i}\left(t\right)\right)}^{2}\sum_{i=1}^{N}\sum_{t=1}^{T}{\left(L^{k}x_{i}\left(t+s\right)\right)}^{2}}}\\ L^{0}x_{i}\left(t\right)=x_{i}\left(t\right),L^{l}x_{i}\left(t\right)=\sum_{j=1}^{N}\omega_{ij}^{l}x_{j}\left(t\right),\sum_{j=1}^{N}\omega_{ij}^{l}=1 \end{array}$$
    where $\rho_{lk}\left(s\right)$ is ST-ACF. t is the statistical period, and s is the time delay. $L^{l}$ is the l-order spatial delay operator of the road network. $L^{l}x_{i}\left(t\right)$ is the comprehensive observation value of traffic with all l-order adjacent roads of the TR. $\omega_{ij}^{l}$ is the weight of l-order adjacent roads.
  (b) CCF
    (2) $$\rho_{XY}\left(s\right)=\frac{E\left[\left(x\left(t\right)-\mu_{x}\right)\left(y\left(t+s\right)-\mu_{y}\right)\right]}{\sqrt{\sigma_{X}^{2}\sigma_{Y}^{2}}}$$
    where $\rho_{XY}\left(s\right)$ is the correlation value of data series. x(t) and y(t+s) are the corresponding values, $\mu_{x}$ and $\mu_{y}$ are their average values, and $\sigma_{X}$ and $\sigma_{Y}$ are their standard deviations.

The first two indexes can calculate the correlation considering only one dimension (time or space). The ST-ACF reflects the overall correlation between different orders of the road network. The CCF can calculate the correlation between two single roads. It is worth noting that most of these methods are based on the Pearson coefficient or its variant structures [35]. Since only several statistical indicators, mean and variance, are used to calculate the correlation of different data series, this method has high generalization ability and has been applied to many fields, including traffic state analysis. However, a fact that cannot be ignored is that the Pearson coefficient based on statistical theory can only reveal the correlation between calculated objects at the data level rather than the physical level.

At the same time, based on the advantages of current technology development, many data collection and calculation models have been proposed. The heterogeneous sensor networks and distributed computing system proposed in related research [36,37,38] could provide new help for the collection and analysis of road network traffic data, and help to deeply mine the road network traffic correlation.

Therefore, based on the traffic dynamic theory and the structural characteristics of urban road networks, this paper innovatively proposes a novel approach calculation model of STC.

## 3. Physical Parameters for STC Calculation

**Figure 1:**
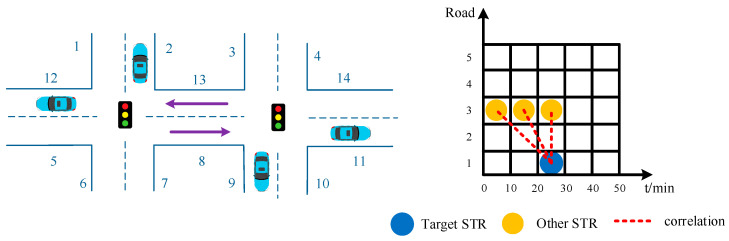
Schematic of a traffic road network.

Specifically, STC is the correlation between the traffic statuses of different roads in different time periods. Without loss of generality, a typical urban road network can be illustrated, as shown in the left subgraph in Figure 1. Note that the traffic state must be treated from the perspective of time and space. The traffic state may correspond to a spatial–temporal network composed of different spatial–temporal regions (STRs), as shown in the right subgraph of Figure 1. The ordinate and abscissa represent different roads and different times, respectively. The STR is the traffic data collected in different time periods. The STC is the correlation between two different STRs, as shown in Figure 1. For convenience, we call the road affected by other roads the target road (TR) in this paper.

Considering that the traffic flow has the characteristics of dynamic update, the influence time of the target road affected by the historical traffic state of other roads must be limited. Therefore, in Section 3.1, the parameter of traffic complete influence time and its calculation method is proposed, which is based on the transmission of traffic flow and traffic waves, to measure the influence time of the target road affected by the traffic state of other roads in the historical period.

Moreover, considering the characteristics of urban road networks with multiple branches, this paper proposes the parameter of traffic correlation strength calculated by the flow ratio to measure the correlation intensity of different roads to the same target road based on the diversion combined mechanism of intersections in Section 3.2.

### 3.1. Traffic Complete Influence Time and Its Calculation Method

With the continuous flow of newly entering traffic, the composition of the traffic flow on the TR changes with time. Assume traffic condition of two roads, road i and road j, at different sampling times, let road j is upstream of road i. Assume that all the traffic on road i during a period is generated by road j. Then, the traffic state of road i in the sampling period (A) might be very similar to that of road j in the sampling period (C). However, in the sampling period (B), all the vehicles from road j in the sampling period (C) drive out of road i. Therefore, in fact, there is no correlation between these two STRs. Thus, it leads to a very important characteristic, that is, the influence time of traffic state in different spatial–temporal regions (STRs) is limited.

#### 3.1.1. The Traffic Complete Influence Time (TCIT)

The specified correlation refers to the correlation between two traffic data for the same length. Suppose the correlation between the traffic state of road j in time period $\left(\tau_{j,1},\tau_{j,n}\right)$ and that of road i in time period $\left(\tau_{i,1},\tau_{i,n}\right)$ need to be calculated. The time delay between them is $\tau_{i,1}-\tau_{j,1}\geq 0$.

Theoretically, as long as there is a connection between the two roads, the traffic state of road j will affect the traffic state of road i at every moment. However, due to the existence of road length, it takes time for the influence to transfer to or out of road i. Therefore, the influence of road j on road i has time delay characteristics at each time, and the time delay may be different. That is, the maximum influence time of road j on road i will be different when the starting time is different.

Therefore, to determine the maximum influence time of the traffic state of road j in time period $\left(\tau_{j,1},\tau_{j,n}\right)$ on that of road i in time period $\left(\tau_{i,1},\tau_{i,n}\right)$, it is necessary to analyse the influence time of every time in this period.

In period $\left(\tau_{j,1},\tau_{j,n}\right)$, the maximum influence time of road j on road i starting from time $\tau_{j,s}$ was called as the local influence time (LIT), that is, $\left\{t_{j,i}\left(\tau_{j,s}\right)|1\leq s\leq n\right\}$. We call the maximum value of the LITs in time sequence $\left[t_{j,i}\left(\tau_{j,1}\right),\cdots ,t_{j,i}\left(\tau_{j,n}\right)\right]$ the traffic complete influence time (TCIT) from road j to road i in time period $\left(\tau_{j,1},\tau_{j,n}\right)$, that is, $\xi_{j,i}\left(\tau_{j,1}\right)$. Once the time exceeds the TCIT, with the addition of new information, the influence of road j on road i generated in period $\left(\tau_{j,1},\tau_{j,n}\right)$ is expected to gradually change and eventually disappear. Therefore, determining the local influence time is a critical aspect in the analysis.

#### 3.1.2. TCIT Calculation Method

To calculate the LIT, it is necessary to explore the causes of the traffic state change. The LWR model is also called the kinematic wave model since it describes the evolution of traffic dynamics as a combination of characteristic, shock, and rarefaction waves. These waves result from the movements of vehicles [39]. As the fundamental diagram, the LWR model describes the average traffic dynamics. This model mainly analyses the changes in traffic flow, speed and density with spatial location and time in the macro state.

According to the theory of the LWR model, there are two main ways to change the traffic state: traffic flow and traffic wave. Only when the traffic flow or traffic wave exists in the target road will it affect its traffic state. Therefore, the time of the traffic flow and traffic wave passing out of the target road was calculated to measure the LIT. Then, the TCIT to the target road in the whole time series is obtained by calculating the maximum value of LITs at different times.

The influence principle of traffic flow and traffic waves on traffic states and the detailed calculation process of TCIT are as follows.

First, according to the traffic dynamic model, the traffic between any two points on the road follows the law of conservation of traffic flow:

(3) $$\frac{\partial q}{\partial x}+\frac{\partial k}{\partial t}=g\left(x,t\right)$$

where q is the traffic flow, k is the traffic density, x is the road location variable, and t is the time variable. g(x,t) is the vehicle generation (or departure) rate. In the conservation equation, the traffic flow and speed can be regarded as a function of density, and then the dynamic equation of traffic flow can be derived:

(4) $$\frac{dv}{dt}=-k{\left(\frac{dv}{dk}\right)}^{2}\frac{\partial k}{\partial x}$$

where v is the traffic speed. Formula (4) shows that the average acceleration of vehicles, $\frac{dv}{dt}$, depends on the density gradient of traffic flow, $\frac{\partial k}{\partial x}$. If $\frac{\partial k}{\partial x}>0$, that is, when the traffic density tends to increase, $\frac{dv}{dt}<0$, the vehicle decelerates; if $\frac{\partial k}{\partial x}<0$, that is, when the traffic density tends to decrease, $\frac{dv}{dt}>0$, the vehicle accelerates.

This dynamic equation proved that the traffic flow has a direct impact on the road traffic state. Therefore, the calculation method for LIT caused by the transmission of traffic flow from road j starting from time $\tau_{j}$ to road i was:

(5) $$t_{j,i}\left(\tau_{j},1\right)=\min\left\{\tau |L_{v}\left(\tau_{j},\tau \right)\geq L_{j,i}\right\}$$

where $t_{j,i}\left(\tau_{j},1\right)$ represents the end time of the impact of adjacent road j on road i from time $\tau_{j}$. $L_{j,i}$ is the distance between the two roads, and $L_{j,i}=l_{j}+l_{i}$, $l_{j}$ and $l_{i}$ are the lengths of roads j and i, respectively. $L_{v}\left(\tau_{j},\tau \right)$ is the transmission distance of the traffic flow from time $\tau_{j}$ to time τ, calculated as:

(6) $$L_{v}\left(\tau_{j},\tau \right)=\int_{\tau_{j}}^{\tau_{1}}v_{j}\left(t\right)dt+\int_{\tau_{1}}^{\tau }v_{i}\left(t\right)dt$$

where $\tau_{1}$ represents the moment at which all the traffic flows out of road j. $v_{i}$ is the average traffic speed of road i.

Second, according to LWR theory, the problem of solving the traffic state of two roads or two locations satisfies the condition of the Riemann problem [40]. When the traffic density of the road changes, a traffic wave similar to a “shock wave” will be generated and transmitted between the roads. There will be a gradual change of traffic state. Traffic wave theory provides a good explanation for the formation and development of the macrotraffic state of roads. For example, Refs. [41,42,43] carried out relevant research on road congestion, which estimates the queue length by predicting the propagation speed of traffic waves and then characterizes the traffic state of the road. Inspired by this, this paper proposed a method to calculate the influence time between different roads through the transmission of traffic waves.

Considering the directivity of traffic wave transmission, the positive direction of the traffic wave was specified as the same as the direction of its traffic flow in this work. If the traffic wave disappears or reverses direction in the entire transmission process, the transmission of the current traffic wave is considered to be completed.

Therefore, the calculation method of LIT caused by the transmission of traffic waves from road j starting from time $\tau_{j}$ to road i could be:

(7) $$\begin{array}{l} t_{j,i}\left(\tau_{j},2\right)=\min\left\{\tau_{1},\tau_{2}\right\}\\ \tau_{1}=\min\left\{\tau |L_{\omega }\left(\tau_{j},\tau \right)\geq L_{j,i}\right\}\\ \tau_{2}=\min\left\{\tau |\omega \left(\tau \right)\omega \left(\tau -1\right)\leq 0\right\} \end{array}$$

where $L_{\omega }\left(\tau_{j},\tau \right)$ is the transmission distance of the traffic wave from time $\tau_{j}$ to time τ, which can be expressed as

(8) $$L_{\omega }\left(\tau_{j},\tau \right)=\int_{\tau_{j}}^{\tau }\omega \left(t\right)dt$$

where ω is the speed of the traffic wave. According to the classical first-order steady-state LWR model, the traffic wave speed can be calculated as:

(9) $$\omega =\frac{q_{j}-q_{i}}{k_{j}-k_{i}}$$

where $q_{j}$ and $q_{i}$ are the traffic flows of roads j and i, respectively, and $k_{j}$ and $k_{i}$ are their traffic densities.

However, when considering the possibility of traffic flow interruption between two roads in the transmission of traffic waves, it is necessary to modify Formula (8).

(1) If the traffic wave is not transmitted out of road j when the interruption occurs, the transmission will terminate. $L_{\omega }\left(\tau_{j},\tau \right)$ can be calculated as:
  (10) $$L_{\omega }\left(\tau_{j},\tau \right)=\int_{\tau_{j}}^{\tau_{end}}\omega_{j}\left(t\right)dt\;\;\;\;\;\;\;\;\;\;\;\;\tau_{end}=\min\left(\tau_{0},\tau_{2}\right)$$
  where $\tau_{0}$ is the moment when the traffic flow between roads becomes 0. $\tau_{2}$ is the moment when the traffic wave passes out of road j. $\omega_{j}$ is the wave speed on road j. Due to the interruption of the traffic flow, the LIT is $t_{j,i}\left(\tau_{j},2\right)=\;\;\tau_{end}$.
(2) If the traffic wave has passed through road j but not through road i when the interruption occurs, the whole process will be divided into two stages. Before the interruption, the traffic wave is transmitted according to speed $\omega_{j}$. After the interruption, road j is equivalent to a road with a traffic flow and density of 0. Therefore, $L_{\omega }\left(\tau_{j},\tau \right)$ at this stage can be calculated as:
  (11) $$L_{\omega }\left(\tau_{j},\tau \right)=\int_{\tau_{j}}^{\tau_{0}}\omega_{j}\left(t\right)dt+\int_{\tau_{0}}^{\tau }\omega_{i}\left(t\right)dt$$
  where $\tau_{0}\geq \tau_{2}$. According to the LWR model, the speed is calculated as:
  (12) $$\omega_{i}\left(x\right)=\frac{\left|q_{i}-0\right|}{\left|k_{i}-0\right|}$$
  where $\omega_{i}$ is the transmission speed of the traffic wave on road i.

Finally, note that for the upstream road, both the traffic flows and traffic waves may enter the downstream road. Therefore, combining Formulas (5) and (7), the LIT starting at time $\tau_{j}$ can be calculated as follows:

(1) When the TR is the downstream road,
  (13) $$t_{j,i}\left(\tau_{j}\right)=\min\left\{t_{j,i}\left(\tau_{j},1\right),t_{j,i}\left(\tau_{j},2\right)\right\}$$
(2) When the TR is the upstream road,
  (14) $$t_{j,i}\left(\tau_{j}\right)=t_{j,i}\left(\tau_{j},2\right)$$

Therefore, the TCIT of period $\left(\tau_{j,1},\tau_{j,n}\right)$ can be calculated as follows:

(15) $$\xi_{j,i}\left(\tau_{j,1}\right)=\max\left(t_{j,i}\left(t\right)\right),t\in \left(\begin{array}{cccc} \tau_{j,1} & \tau_{j,2} & \cdots & \tau_{j,n} \end{array}\right)$$

### 3.2. Traffic Correlation Strength and Its Calculation Method

**Figure 2:**
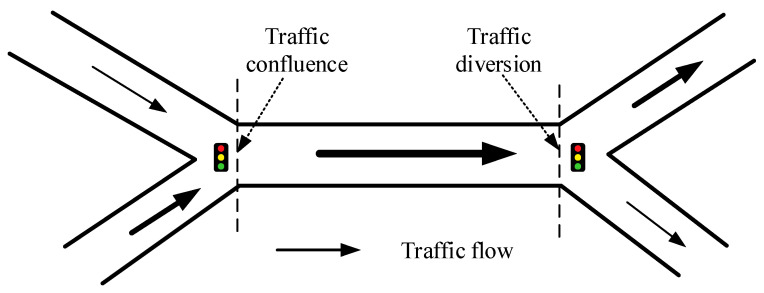
Schematic diagram of the diversion and confluence mechanism of urban road intersections.

As shown in Figure 2, the multibranch structure with different guidance directions determines that the intersection has the function of diversion and confluence [44]. The traffic flow of different upstream roads to the downstream will be different. There is no doubt that the most direct influence of this characteristic is that different roads may have different impacts on the same road. Therefore, it is necessary to consider this when calculating the STC of different roads on the target road. To this end, first, the instantaneous correlation strength between two roads is calculated. Then, on this basis, the traffic correlation strength (TCS) between the two STRs in the whole time series is calculated.

#### 3.2.1. Instantaneous Correlation Strength

In a certain period, as more traffic flows are transferred between two roads, the relationship between them will be more significant. To measure the influence intensity between two roads, this paper introduces the traffic flow ratio to determine the correlation strength between the roads. The traffic flow ratio is calculated as:

(16) $$\alpha_{j,i}\left(\tau \right)=\left\{ \begin{array}{l} \frac{q_{j,i}\left(\tau \right)}{q_{i}\left(\tau \right)}\;\;\;\;\;\;\;\;\;\;\;\;\;\;\;\;\;j\;is\;the\;upstream\\ \frac{q_{i,j}\left(\tau \right)}{q_{j}\left(\tau \right)}\;\;\;\;\;\;\;\;\;\;\;\;\;\;\;\;\;i\;is\;the\;upstream \end{array}\right.$$

where $\alpha_{j,i}\left(\tau \right)$ is the traffic flow ratio at time τ between road i and road j. $q_{j,i}\left(\tau \right)$ is the traffic flow from road j to road i at time τ, and $q_{i}\left(\tau \right)$ is the total traffic flow at time τ to road i from its adjacent roads.

Due to the different relative positions of the road, the direction of the influence between two roads will be different. Therefore, this paper evaluates whether road j influences road i according to its influence direction. Based on the different ways of influence, the influence direction will be calculated as:

(17) $$\beta_{j,i}\left(\tau \right)=\left\{ \begin{array}{l} \left\{ \begin{array}{l} 1\;\;\;\;\;\;\;\;\;\;\;\;if\;\;\;\;\alpha_{j,i}\left(\tau \right)\neq 0\\ 0\;\;\;\;\;\;\;\;\;\;\;\;if\;\;\;\;\alpha_{j,i}\left(\tau \right)=0 \end{array}\right.\;\;\;\;\;\;\;\;\;\;\;\;j\;is\;the\;upstream\\ \left\{ \begin{array}{l} 1\;\;\;\;\;\;\;\;\;\;\;\;if\;\;\;\;\omega_{j}\left(\tau \right)>0\\ 0\;\;\;\;\;\;\;\;\;\;\;\;if\;\;\;\;\omega_{j}\left(\tau \right)\leq 0 \end{array}\right.\;\;\;\;\;\;\;\;\;\;\;\;\;\;i\;is\;the\;upstream \end{array}\right.$$

where $\beta_{j,i}\left(\tau \right)$ is used to assess whether road j has a direct impact on road i at time τ.

The product $\delta_{j,i}\left(\tau \right)=\alpha_{j,i}\left(\tau \right)\beta_{j,i}\left(\tau \right)$ is considered to measure the instantaneous correlation strength (ICS) between two adjacent roads at time τ. Generally, a larger value of $\delta_{j,i}\left(\tau \right)$ corresponds to a stronger correlation.

Note that the correlation between two roads will not change significantly in two adjacent periods because the traffic flow is continuous. Thus, $\alpha_{j,i}\left(\tau \right)=0$ does not mean that no correlation exists between the roads. For example, even if there is no traffic flow between the congested road and its upstream road, a strong correlation may exist between them. Thus, it is necessary to consider the traffic state of roads when calculating the correlation strength. $\eta_{i}\left(\tau \right)$ is adopted to express the state of road i at sampling time τ.

(18) $$\eta_{i}\left(\tau \right)=\{\begin{array}{cc} 1 & if\;\;q_{i}\left(\tau \right)=0\\ 2 & \;\;\;\;\;\;\;\;\;\;\;\;\;\;\;\;\;\;\;\;if\;\;0<q_{i}\left(\tau \right)\&v_{i}\left(\tau \right)\neq 0\\ 3 & if\;\;v_{i}\left(\tau \right)=0 \end{array}$$

Thus, without losing generality, supposing traffic flows from road j to road i, the calculation of instantaneous correlation strength could then be modified, as shown in Listing 1.

**Listing 1:** Progress for the calculation of the Instantaneous Correlation Strength.

**if** $q_{j,i}\left(\tau \right)=0$ **//if there is no traffic flow between two adjacent roads**
**if** $\eta_{i}\left(\tau \right)=1$ **or** $\eta_{j}\left(\tau \right)=1$ **//if at least one of them is empty**
$\delta_{j,i}\left(\tau \right)=0$ **//then the ICS between them is 0**
**else** **//if both roads are not empty**
**if** $q_{i}\left(\tau \right)=0$ **//if the upstream road i is congested**
**if** $q_{i}\left(\tau -1\right)=0$ **//and it is also in the congestion state at the previous time step**
$\delta_{j,i}\left(\tau \right)=\delta_{prev}$ **//then the ICS remains the same value during the congestion period**
**else** **//Otherwise, if the upstream road i was not congested at the**
**//previous time step**
$\delta_{j,i}\left(\tau \right)=\alpha_{j,i}\left(\tau -1\right)\beta_{j,i}\left(\tau -1\right)$**//calculate the ICS according to the data in the**
**//previous sampling time**
$\delta_{prev}=\alpha_{j,i}\left(\tau \right)\beta_{j,i}\left(\tau \right)$ **//update the ICS value during this congestion period**
**//for later use**
**end**
**else** **//if road i is not congested**
$\delta_{j,i}\left(\tau \right)=\alpha_{j,i}\left(\tau \right)\beta_{j,i}\left(\tau \right)$ **//calculate the current ICS directly**
**end**
**end**
**else** **//if there exists traffic flow between two roads**
$\delta_{j,i}\left(\tau \right)=\alpha_{j,i}\left(\tau \right)\beta_{j,i}\left(\tau \right)$ **//calculate the current ICS directly**
**end**

#### 3.2.2. TCS Calculation Method

The instantaneous correlation strength only reflects the correlation strength of two roads at a certain moment, while the calculation of STC is for the whole time series. In addition, with the continuous updating of time, the traffic state in the specified STR has different effects on that of the target road with different time periods. Therefore, it is necessary to consider the influence of time delay when calculating the TCS.

Assume that the TCIT from the traffic state of road j with time period $\left(\tau_{j,1},\tau_{j,n}\right)$ to that of road i in time period $\left(\tau_{i,1},\tau_{i,n}\right)$ is $\xi_{j,i}\left(\tau_{j,1}\right)$. As the time period of road i slowly exceeds $\xi_{j,i}\left(\tau_{j,1}\right)$, with the influx of the traffic flow from other roads, the TCS owing to road j with time period $\left(\tau_{j,1},\tau_{j,n}\right)$ will gradually decrease. Therefore, the TCS of road j on i in different periods could be determined as:

If $\tau_{i,n}\leq \xi_{j,i}\left(\tau_{j,1}\right)$,

(19) $$f_{j,i}\left(\tau_{j,1},\tau_{i,1}\right)=\lambda_{j}$$

Otherwise,

(20) $$f_{j,i}\left(\tau_{j,1},\tau_{i,1}\right)=\left\{ \begin{array}{l} \lambda_{j}\gamma_{1}\gamma_{2}\;\;\;\;\;\;\;\;\;\;\;\;\;\;\;\;\;\;\;\;\;\;\;\;\;\;\;\;\;if\;\;\;\xi_{j,i}\left(\tau_{j,1}\right)-\tau_{i,1}>0\\ 0\;\;\;\;\;\;\;\;\;\;\;\;\;\;\;\;\;\;\;\;\;\;\;\;\;\;\;\;\;\;\;\;\;\;\;\;\;\;\;otherwise \end{array}\right.$$

(21) $$\lambda_{j}=\frac{1}{\left(\tau_{j,n}-\tau_{j,1}\right)}\sum_{\tau =\tau_{j,1}}^{\tau_{j,n}}\delta_{j,i}\left(\tau \right),\;\;\;\gamma_{1}=\frac{\xi_{j,i}\left(\tau_{j,1}\right)-\tau_{i,1}}{\xi_{j,i}\left(\tau_{j,1}\right)-\tau_{j,1}},\;\;\;\gamma_{2}=\frac{\sum_{\tau =\tau_{i,1}}^{\xi_{j,i}\left(\tau_{j,1}\right)}\delta_{j,i}\left(\tau \right)}{\sum_{\tau =\tau_{i,1}}^{\tau_{i,n}}\delta_{j,i}\left(\tau \right)}\;$$

where $f_{j,i}\left(\tau_{j,1},\tau_{i,1}\right)$ is the TCS between the traffic state of road j, with $\tau_{j,1}$ as the starting time, and the traffic state of road i, with $\tau_{i,1}$ as the starting time. $\lambda_{j}\;$ is the initial influence intensity within the TCIT of road j. $\;\gamma_{1}\;$ and $\;\gamma_{2}$ represent the proportion of time and strength for the influence of road j on road i. These two parameters reflect the impact of the time delay on the influence intensity. A larger traffic flow ratio corresponds to a greater initial influence intensity. A greater time delay corresponds to a smaller influence intensity.

## 4. STC Calculation Method for Urban Roads

Compared with adjacent roads with direct impact, there is no direct correlation between the traffic statuses of two non-adjacent roads. In view of this, we creatively propose a method to calculate the correlation between roads according to road adjacency.

First, according to the analysis presented above, the calculation method of the STC between two adjacent roads was established in Section 4.1. Then, based on this, the calculation method between two non-adjacent roads is established in Section 4.2. Finally, we summarize the overall calculation process in Section 4.3.

### 4.1. Calculation Method for Adjacent Roads

Assume that the traffic data of the TR (road i) and its adjacent road j can be expressed as follows:

(22) $$\begin{array}{l} d_{i}\left(\tau_{i,1}\right)=\left(\begin{array}{cccc} x_{\tau_{i,1}} & x_{\tau_{i,2}} & \cdots & x_{\tau_{i,n}} \end{array}\right)\\ d_{j}\left(\tau_{j,1}\right)=\left(\begin{array}{cccc} y_{\tau_{j,1}} & y_{\tau_{j,2}} & \cdots & y_{\tau_{j,n}} \end{array}\right) \end{array}$$

where $x_{\tau_{i,1}}$ and $y_{\tau_{j,1}}$ are the traffic data, for example, the traffic speed, with times of $\tau_{i,1}$ and $\tau_{j,1}$, respectively. $d_{j}\left(\tau_{j,1}\right)$ and $d_{i}\left(\tau_{i,1}\right)$ are the traffic data sequences of roads j and i. Here, without loss of generality, assume that $\tau_{j,1}\leq \tau_{i,1}$. The STC between the roads can be calculated as:

(23) $$\begin{array}{l} r_{j,i}\left(\tau_{j,1},\tau_{i,1}\right)=\rho_{j,i}\left(\tau_{j,1},\tau_{i,1}\right)f_{j,i}\left(\tau_{j,1},\tau_{i,1}\right)\\ \rho_{j,i}\left(\tau_{j,1},\tau_{i,1}\right)=\frac{E\left[\left(d_{j}\left(\tau_{j,1}\right)-\mu_{j}\right)\left(d_{i}\left(\tau_{i,1}\right)-\mu_{i}\right)\right]}{\sigma_{j}\sigma_{i}} \end{array}$$

where $\mu_{i}$ and $\sigma_{i}$ are the average values and standard deviations, respectively, of data $d_{i}\left(\tau_{i,1}\right)$. $r_{j,i}\left(\tau_{j,1},\tau_{i,1}\right)$ is the correlation between the traffic state of road j starting from time $\tau_{j,1}$ and that of road i starting from time $\tau_{i,1}$.

Note that the calculation includes two parts: $\rho_{j,i}\left(\tau_{j,1},\tau_{i,1}\right)$ and $f_{j,i}\left(\tau_{j,1},\tau_{i,1}\right)$. The former is derived from the cross-correlation function (CCF) of statistical theory, which reveals the similarity between two traffic data and is used to measure the consistency of the change trend of two traffic statuses. The latter reveals the degree of internal correlation between the traffic states.

Therefore, this calculation method considers not only the similarity between the traffic information (as an external performance feature) but also the impact of different traffic flow proportions and time delays (as an internal feature). It introduces physical meaning to the calculation of STC between adjacent roads.

### 4.2. Calculation Method for Non-Adjacent Roads

Different from adjacent roads, there is no direct influence between non-adjacent roads. However, due to the connectivity of roads, an exchange of traffic information might occur between any two non-adjacent roads [45]. The traffic flow between these roads is realized by the transmission path between the roads [46]. Hence, the correlation between each pair of adjacent roads in the path will affect the overall correlation between the two non-adjacent roads. The traditional calculation method does not consider the physical structure of the road network and ignores the actual transmission characteristics of traffic flow. As a result, regardless of how far away the two roads are, they are directly adjacent by default in the whole calculation process.

**Figure 3:**
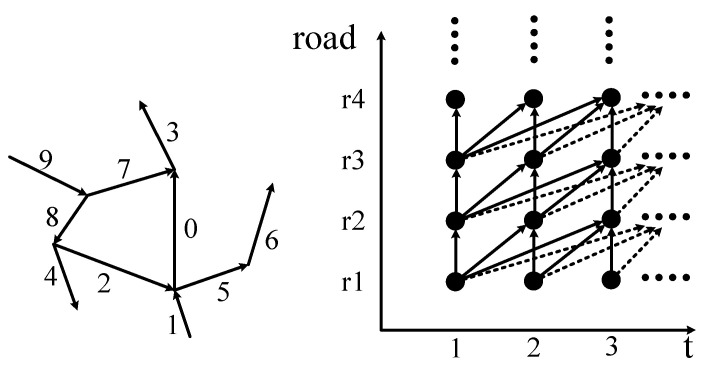
Schematic of the correlation of the road network.

For a deeper analysis, a schematic of the road network topology was constructed in Figure 3. The left diagram shows a road network structure, and the right diagram depicts the relationship of the STRs. The abscissa and ordinate represent different starting times and roads, respectively. The arrows represent the connection between two STRs. As shown in the right subgraph of Figure 3, each road will have an impact on the current and future traffic states of its adjacent roads. The specific impact time is calculated according to different traffic conditions.

Through analysis, it is worth noting that the influence between traffic states of non-adjacent roads could be realized on the basis of adjacent roads in the path between them. For example, if there is a traffic jam on road 6 in Figure 3, the traffic state of road 5 will change first, and the change of traffic state of road 5 will then affect the traffic state of road 1 or road 2. Therefore, based on the continuity in time and transitivity in space, this paper proposed a calculation method for the correlation between any two non-adjacent roads by considering the transitivity in the path between them. Without loss of generality, we take the correlation calculation of road 9 at time 1 to the TR (road 0) at time 3 as an example to illustrate the process.

**Figure 4:**
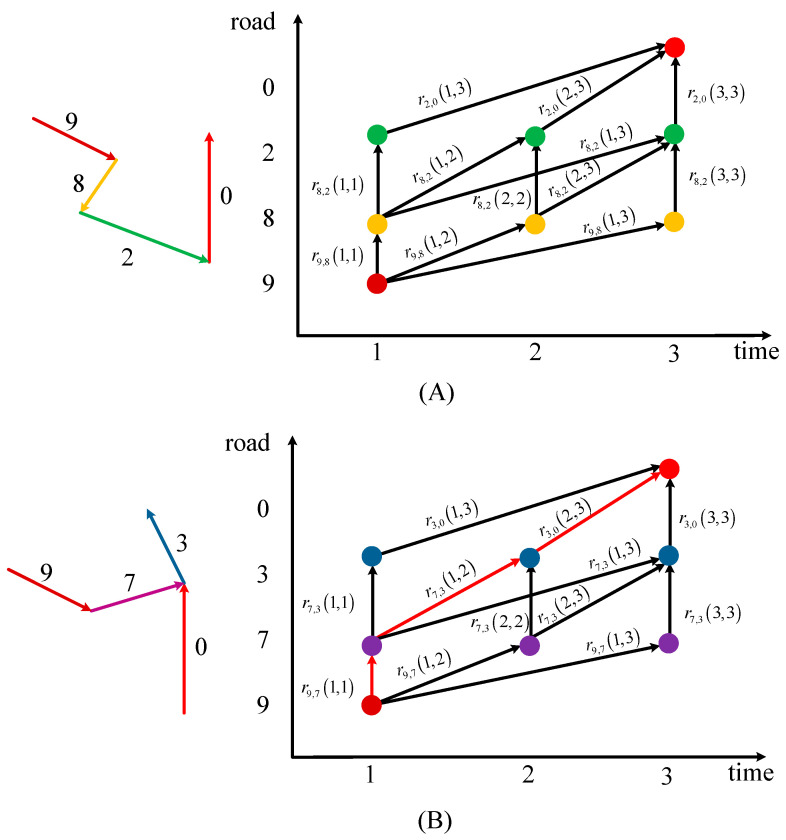
Two different transmission paths of road 9 to the TR (road 0) extracted from the road network.

Two main influence paths exist, as shown in Figure 4A,B: the left figures show the path extracted from the road network, and the right figures show the transmission diagram of correlation. Each dot represents an STR, and the arrow represents the correlation between the two STRs. Even if the path is determined, many transmission paths exist for the correlation between the STRs due to the existence of time delays. Taking the calculation of $r_{9,0}\left(1,3\right)$ in Figure 4B as an example, there are six influence transmission paths:

(24) $$\begin{array}{l} r_{9,7}\left(1,1\right)\to r_{7,3}\left(1,1\right)\to r_{3,0}\left(1,3\right)\\ r_{9,7}\left(1,1\right)\to r_{7,3}\left(1,2\right)\to r_{3,0}\left(2,3\right)\\ r_{9,7}\left(1,1\right)\to r_{7,3}\left(1,3\right)\to r_{3,0}\left(3,3\right)\\ r_{9,7}\left(1,2\right)\to r_{7,3}\left(2,2\right)\to r_{3,0}\left(2,3\right)\\ r_{9,7}\left(1,2\right)\to r_{7,3}\left(2,3\right)\to r_{3,0}\left(3,3\right)\\ r_{9,7}\left(1,3\right)\to r_{7,3}\left(3,3\right)\to r_{3,0}\left(3,3\right) \end{array}$$

Take the path of the red arrows in Figure 4B as an example, that is, $r_{9,7}\left(1,1\right)\to r_{7,3}\left(1,2\right)\to r_{3,0}\left(2,3\right)$. Considering the transfer characteristics of traffic, a calculation method for the correlation between road 1 and road 3 was proposed as:

(25) $$r_{9,0}\left(1,3\right)=r_{9,7}\left(1,1\right)r_{7,3}\left(1,2\right)r_{3,0}\left(2,3\right)$$

As multiple correlations may be calculated using different transmission paths, to express the correlation more clearly, this paper considers the maximum value as the correlation between the roads. To automatically search the possible path and calculate this maximum value, this paper builds the algorithm based on the idea of the dynamic programming (DP) algorithm. Thus, the correlation calculation between non-adjacent roads can be transformed into an iterative calculation of the STC between adjacent roads. The objective function of this algorithm is:

(26) $$\begin{array}{l} J(g_{f},\tau_{f},\tau_{i})=\max\left(\left(J(g,\tau_{k},\tau_{i})r_{g_{f},g}\left(\tau_{f},\tau_{k}\right)\right)\right)\\ \tau_{f}\leq \tau_{k}\leq \tau_{i} \end{array}$$

where $J(g_{f},\tau_{f},\tau_{i})$ is the STC between road $g_{f}$ starting at time $\tau_{f}$ and the TR starting at time $\tau_{i}$. $r_{g_{f},g}\left(\tau_{f},\tau_{k}\right)$ is the STC between road $g_{f}$ and its adjacent road g starting with $\tau_{f}$ and $\tau_{k}$, respectively.

This formula incorporates the spatial characteristics to calculate the correlation between the non-adjacent roads. Therefore, compared with those methods that directly calculate the similarity of two traffic state sequences without considering the adjacency of roads, this formula reflects the transmission characteristics of the traffic flow on the road.

### 4.3. Overall Calculation Progress of STC

Considering the connectivity characteristics of the road network, infinite noneffective paths may be generated between two non-adjacent roads. However, most of the transmitted correlations may be extremely low or even 0. Therefore, to avoid the generation of redundant associated paths, the overall calculation progress of STC with the TR as the starting road and the adjacent relationship as the basis was built.

**Figure 5:**
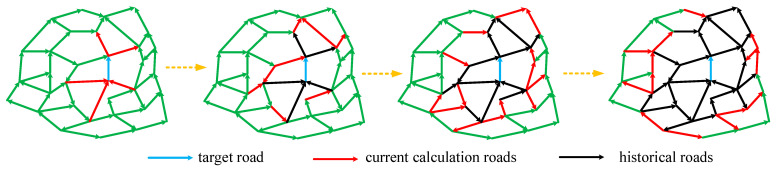
Calculation process of the STC between different roads and the TR.

The pseudocode for the overall calculation of STC for the urban road network is shown in Listing 2. Figure 5 shows the process flow. The blue line represents the TR. The red lines represent the roads that need to be considered in this step. The black lines are the roads that were calculated in the previous step and will not be calculated in subsequent steps.

**Listing 2:** Pseudo code for the overall calculation of STC. Note: where $g_{0}$ is the TR, and $g_{j}$ is the current road. $G_{Link1}$ is the set of adjacent roads of road $g_{j}$. F is the set of historical roads to prevent repeated conflicts in the correlation calculation. G records the next calculated road, which is updated every iteration. $\tau_{0}$ is the starting time of the TR. Based on this analysis, the correlation between different roads and the TR can be obtained.

**Initialisation:**$G\left\{1\right\}\left\{1\right\}=g_{0},num=0,flag=1,F\left\{1\right\}=\emptyset$ $J(g_{0},\tau_{0},\tau_{0})=1$
**Iterative computation**
**while (flag)**
num=num+1 **//record the number of iterations**
k=length(G{num}) **//calculates the number of the current roads involved in this iteration**
n=0
**for** j=1:k
$g_{j}=G\left\{num\right\}\left\{j\right\}$ **//**$g_{j}$ **is the current road**
$G_{Link1}\cap F\left\{num\right\}\left\{j\right\}=\emptyset$ **//remove the historical roads from its adjacent roads**
$G_{Link1}=\left(g_{1},g_{2},\cdots ,g_{m}\right)$
$m=length\left(G_{Link1}\right)$ **//calculate the number of the remaining adjacent roads**
**for** f=1:m **//calculate the correlation between the remaining adjacent roads and the TR**
**for** $\tau_{s}=1:\tau_{0}$ **//**$g_{f}$ **is one of the adjacent roads,** $\tau_{s}$ **is the starting time of road** $g_{f}$
$S(g_{f},\tau_{s},\tau_{0})=\max\left\{J(g_{j},\tau_{k},\tau_{0})r_{g_{f},g_{j}}\left(\tau_{s},\tau_{k}\right)|\tau_{s}\leq \tau_{k}\leq \tau_{0}\right\}$ **//calculated the STC between** $g_{f}$ **and**
**//the TR. See description of Formula (26) for details.**

**if** $S(g_{f},\tau_{s},\tau_{0})>J(g_{f},\tau_{s},\tau_{0})$
$J(g_{f},\tau_{s},\tau_{0})=S(g_{f},\tau_{s},\tau_{0})$ **//select the maximum value as the influence from** $g_{f}$ **with**
**//starting time** $\tau_{s}$ **to the TR with starting time** $\tau_{0}$
**end**
**end**
n=n+1
F{num+1}{n}=[F{num}{j},g] **//update the collection of historical roads for the adjacent roads**
$G\left\{num+1\right\}\left\{n\right\}=G_{Link1}\left(f\right)$ **//update the new collection of current roads**
**end**
**end**
**if** G{num+1}=∅ **//if there is no new calculation**
flag=0 **//the iterative calculation process will be terminated**
**end**
**end**

## 5. Results and Discussion

**Figure 6:**
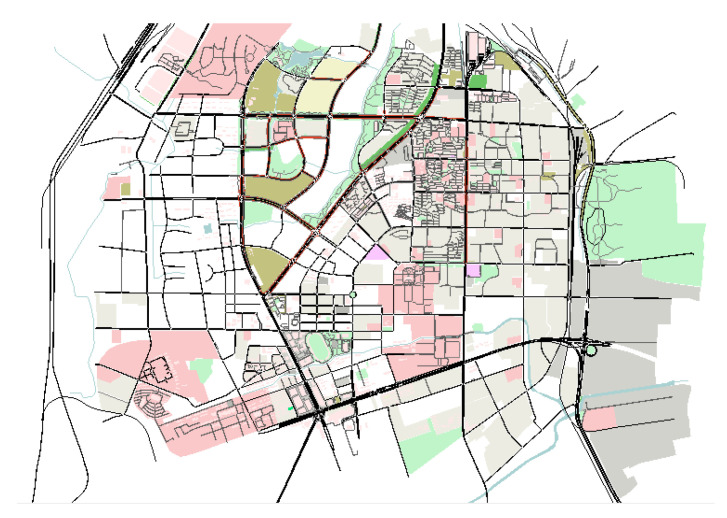
Map of the simulation calculation.

To verify the rationality of the STC calculation method proposed in this paper, a comparative analysis is performed in this section. The verification data are obtained from the operation results of the traffic simulation software Simulation of Urban Mobility (SUMO). The SUMO software, developed by the German Aerospace Centre, is a popular software designed to process large road networks. SUMO can scientifically realize the overall simulation of urban road network traffic with high reliability. The operation map shows an area of Shanghai, China, as shown in Figure 6, and the simulation time is 10,000 s. This map was downloaded from the OpenStreetMap website, including 2167 roads and 5814 intersections with traffic signals. In fact, many advanced simulation models are integrated into SUMO software. Most of the traffic control rules in this paper adopt the default settings of SUMO. The shortest distance is used in vehicle travel path planning. Car following model is based on the Krauss model proposed by German scholar Krauss. The maximum acceleration and maximum deceleration of the simulation vehicles are respectively set by SUMO, that is, 2.6 m/s^2^ and −4.5 m/s^2^. The simulation record parameters include: system time, vehicle speed, location, road network information and traffic light status.

The data employed in macro traffic research are generally data with low and medium sampling frequencies. Thus, to be closer to the real data collection conditions, the research organizes the simulation data with an interval time of 30 s for calculation.

**Figure 7:**
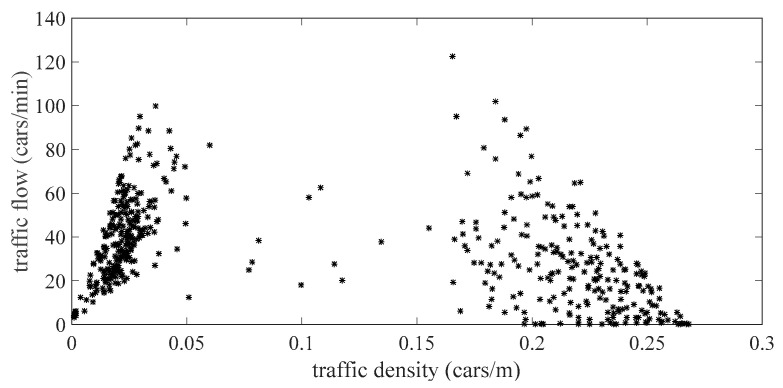
Relationship between traffic density and traffic flow simulated by SUMO in this paper.

The relationship between traffic density and traffic flow of different roads obtained by simulation is shown in Figure 7. The abscissa and ordinate are the traffic density and traffic flow of different roads, respectively. The distribution of the traffic data obtained by simulation is basically consistent with the MFD diagram [21], which conforms to the characteristics of the macro traffic operation law and can provide support for comparative verification.

The length of the data sequence for the STC calculation has is 10 in this paper. To verify the proposed method, two classical models, the original CCF model and the improved CCF model, are utilized to calculate the correlation for comparison. The calculation formulas of the improved CCF is as follows:

(27) $$\begin{array}{l} \rho_{XY}\left(s\right)=\frac{\sum_{t=1}^{T}\left(x_{i}\left(t\right)-{\bar{x}}_{i}\right)\left(L^{k}x_{i}\left(t+s\right)-\bar{L^{k}x_{i}}\right)}{\sqrt{\sum_{t=1}^{T}{\left(x_{i}\left(t\right)-{\bar{x}}_{i}\right)}^{2}\sum_{t=1}^{T}{\left(L^{k}x_{i}\left(t+s\right)-\bar{L^{k}x_{i}}\right)}^{2}}}\\ L^{0}x_{i}\left(t\right)=x_{i}\left(t\right),L^{l}x_{i}\left(t\right)=\sum_{j=1}^{N}\omega_{ij}^{l}x_{j}\left(t\right),\sum_{j=1}^{N}\omega_{ij}^{l}=1 \end{array}$$

where t is the statistical period and s is the time delay. $L^{k}$ is the k-order spatial delay operator of the road network. $L^{k}x_{i}$ is the comprehensive observation value of traffic with all l-order adjacent roads of the TR. $\bar{L^{k}x_{i}}$ is the their average value. $\omega_{ij}^{l}$ is the weight of l-order adjacent roads calculated by distance.

For the convenience of expression, these two models are called CCF1 and CCF2, and the model proposed in this paper is the dynamic correlation function (DCF) in the following content.

To provide a more reliable comparison and highlight the calculation characteristics of different models, this paper chooses three aspects to carry out comparative experiments.

First, a local road network to demonstrate the overall calculation results of the correlation between different roads and the TR in Section 5.1 was extracted. This experiment can highlight the calculation characteristics of different models as a whole level.

Second, the results of different models for adjacent and non-adjacent roads is compared in Section 5.2 and Section 5.3, respectively, to demonstrate the scientific value and rationality of the proposed model.

Finally, multiple analysis of the results of 180 different road networks was performed. The indexes involved in these comparative tests come from the summary of the time delay (Section 5.4), road network topology (Section 5.5) and operation characteristics of roads (Section 5.6), which can indirectly verify the rationality of different models.

### 5.1. Overall Calculation Results of a Road Network

**Figure 8:**
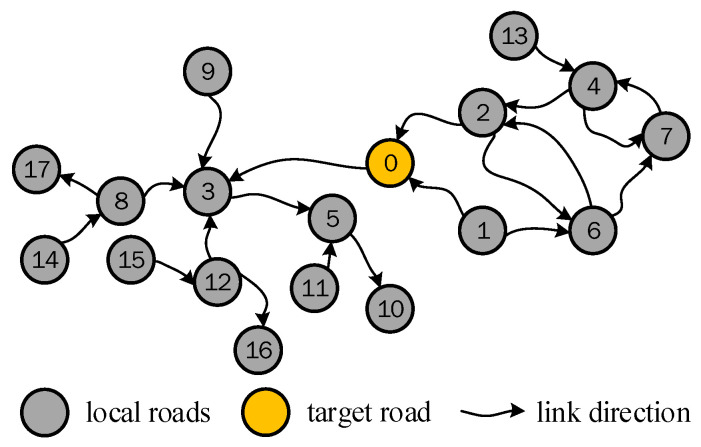
Structure of the road network considered in the test.

**Figure 9:**
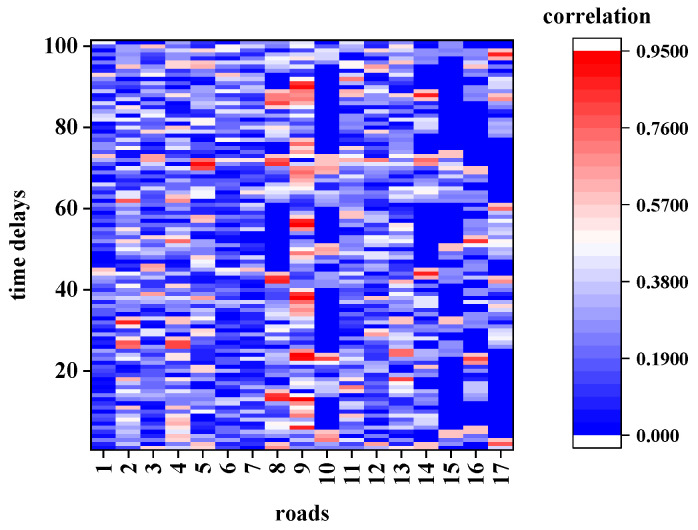
Correlation determined using the original CCF.

**Figure 10:**
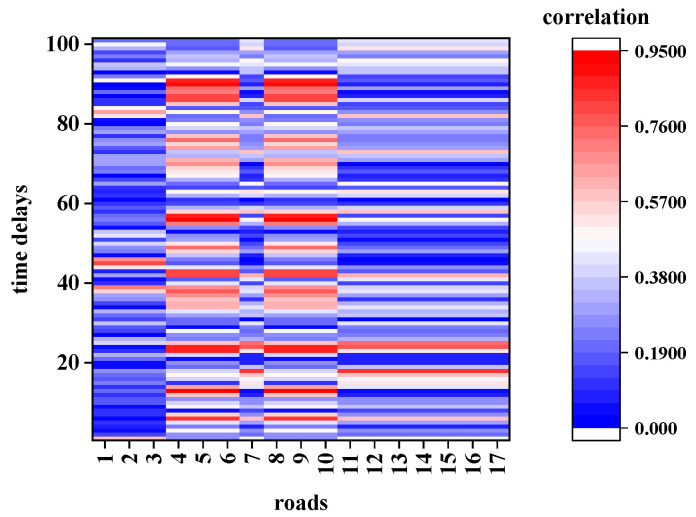
Correlation determined using the improved CCF.

**Figure 11:**
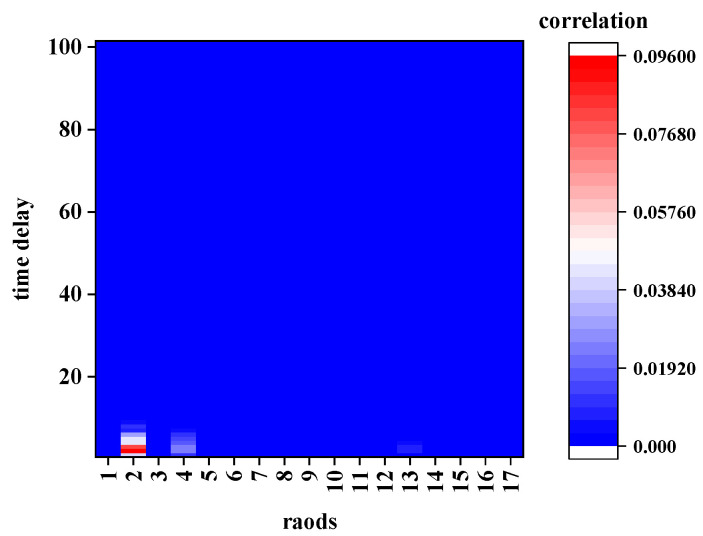
Correlation determined using the DCF.

The extracted road network for this calculation from the map shown in Figure 6, its topological structure is shown in Figure 8, and road 0 is the TR. The average traffic speed data of the TR with a time period of (250–259) was considered as the target STR and calculated the STC with other roads with different time delays. Figure 9 and Figure 10 show the calculation results of CCF1 and CCF2, respectively. Figure 11 shows the calculation results of the proposed DCF method.

According to Figure 9 and Figure 10, the results calculated using the first two methods exhibit the following characteristics:

- In terms of space, the traffic data in the specified period (250–259) of the TR are related to all the roads, and the correlation of different roads is irregular.
- In terms of time, no matter how much the time delays, the other roads could have a significant impact on the traffic state of road 0 in the time period (250–259). Even when the time delays extend over more than 90 sampling intervals (2700 s or 45 min), a strong correlation could be observed, which is not consistent with the actual situation.

However, the calculation result of the DCF shown in Figure 11 shows that:

- The traffic data of the TR in the specified period (250–259) are correlated only with several surrounding roads.
- The correlation decreases to 0 after several time delays.

To highlight the differences among the three models in detail, the calculation was elaborated by considering the conditions of adjacency and non-adjacency in the following contents.

### 5.2. Calculation Results between a Pair of Adjacent Roads

**Figure 12:**
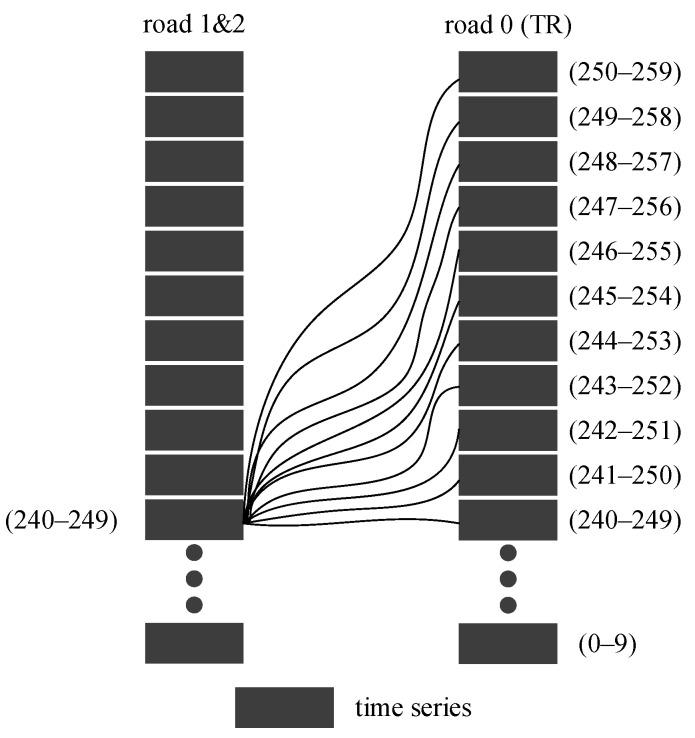
Schematic diagram of the time series between roads participating in the STC calculation and target roads.

To highlight the differences in the calculation between adjacent roads among the three models, this paper considers the calculation of roads 2 and 1 in Figure 8 as an example. The periods selected for the TR are (240–249), (24–250) … (250–259), and the period selected for roads 2 and 1 is (240–249). The time-series position of roads participating in this calculation is shown in Figure 12.

In order to verify the calculation results, we counted the flow ratio of roads 2 and 1 to the TR in the time period (240–259) as shown in Table 1. Table 2 shows the TCS of road 2 to the TR with different time delays, including the TCIT $\xi_{2,0}\left(240\right)$, initial influence intensity $\lambda_{2}$, influence time proportion $\gamma_{1}$, strength proportion $\gamma_{2}$ and TCS $f_{2,0}$.

**Figure 13:**
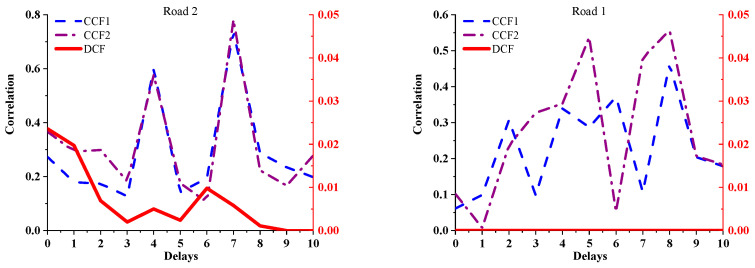
Comparison of correlation between the two roads, as determined using the three algorithms.

The results are shown in Figure 13. The black *Y*-axis on the left shows the reference coordinates of the CCF1 and CCF2 curves, and the red *Y*-axis on the right shows the reference coordinates of the DCF curves. The *X*-axis represents the time delay.

According to Table 1 and Table 2, the following contents can be obtained:

- No vehicles enter the TR during the period (240–249) of road 1. Road 1 does not affect the TR in the period (240–249), as shown in Table 1.
- With the increase in the time delay, the proportion of time and strength that the TR is affected by road 2 in period (240–249) decreases gradually.
- The TCS between TR and road 2 in period (240–249) decreases with an increase in the time delay.

From the calculation results shown in Figure 13, it can be seen that:

- The results of the proposed DCF method are consistent with the content above.
- However, the calculated results of CCF1 and CCF2 do not show any regularity.

Thus, the correlation between two adjacent roads determined by the proposed improved DCF model is more closely related to the actual situation.

### 5.3. Calculation Results between a Pair of Non-Adjacent Roads

**Figure 14:**
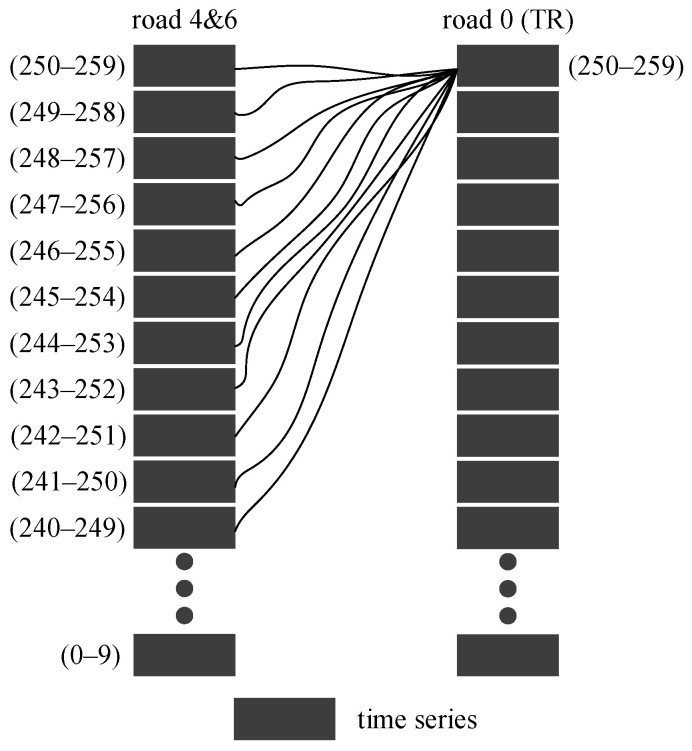
Schematic diagram of the time series between roads participating in the STC calculation and target roads.

To highlight the differences of the three models in the correlation calculation between non-adjacent roads, 11 segments (240–249), (241–250), …, (250–259) were selected for road 4 and road 6 in Figure 8 for the calculation. The time period of the TR is (250–259). The time-series position of roads participating in this calculation is shown in Figure 14.

To make a reliable comparison, a statistical analysis on the traffic flow of roads 4 and 6 with their adjacent roads in the time period (240–259) was performed. The traffic flow ratios are shown in Table 3 and Table 4.

After analysing the contents of Table 1, Table 3 and Table 4, it can be seen that:

- The traffic flow of road 4 in time period (240–259) reaches road 0 through road 2;
- There is no vehicle flow exchange between road 6 and road 2 during this period, but there are some vehicles flowing from road 1 to road 6. However, there is no traffic information exchange between road 1 and road 0 during this period. Therefore, road 6 cannot affect the traffic state of road 0 during this period.

**Figure 15:**
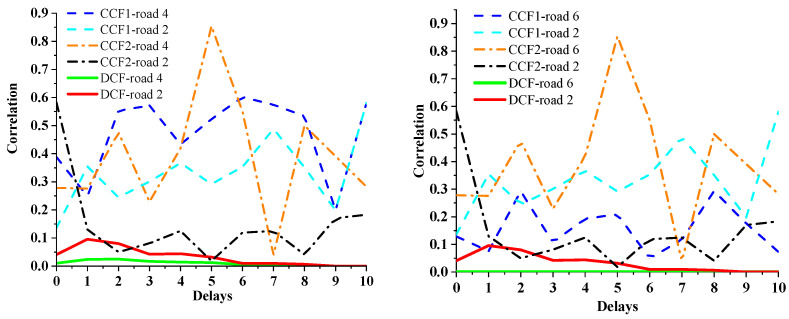
Calculation results for roads 4 and 6.

The calculation results of road 4 and road 6 by the three models are shown in Figure 15.

From the result of Figure 15, it can be seen that:

- The correlation of road 4 calculated by DCF first increases and later decreases to 0 as the time delay increases, and it is less than the influence of road 2 with the same or fewer delay conditions.
- The correlation of road 6 calculated by DCF remains at 0 within different time delays.
- The results of road 4 and road 6 calculated by CCF1 and CCF2 show irregular changes with time delay. Moreover, the calculation results show that the correlation between these roads and the TR has no relationship with the correlation between road 2 and the TR.

However, the correlation between non-adjacent roads will be affected by the correlation of adjacent roads between them, as analysed. As road 2 is directly adjacent to the target road (road 0), it will affect the target road more easily than road 4 and road 6. Therefore, the calculation results of CCF1 and CCF2 are obviously inconsistent with the actual situation. In comparison, the results of the proposed DCF model are more reasonable than those of CCF1 and CCF2.

### 5.4. Relationship between the Average Correlation and Time Delays

**Figure 16:**
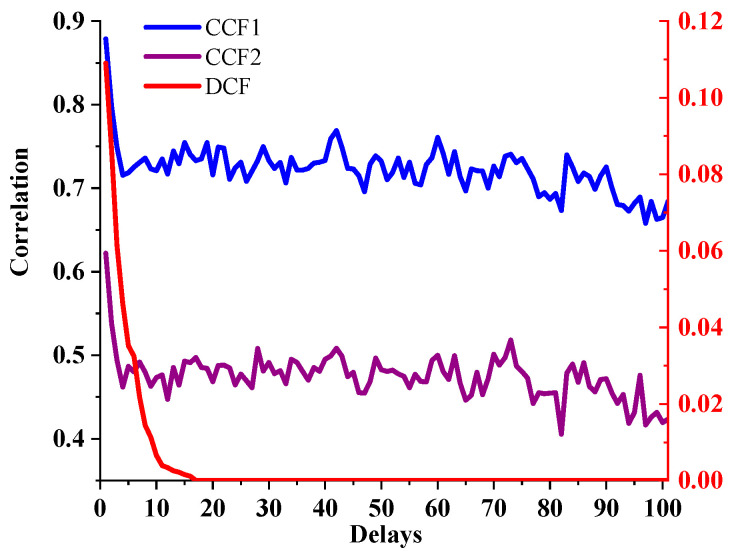
Relationship between the average correlation and time delays.

To clarify the relationship between the time delay and correlation, the average correlation with different time delays was calculated as follows:

(1) Different roads in Figure 6 are selected as the target roads, and then the surrounding road networks centred on these target roads are constructed as the calculation samples.
(2) The average value of the correlation of all samples under different time delays is calculated as:
  (28) $$\mu \left(k,d\right)=\frac{1}{n_{k}}\sum_{j=1}^{n_{k}}r_{j,k}\left(d\right)$$
  (29) $$\bar{\mu }\left(d\right)=\frac{1}{m}\sum_{i=1}^{m}\mu \left(i,d\right)$$
  where $r_{j,k}\left(d\right)$ is the correlation between TR k and its surrounding road j with the time delay d. μ(k,d) is the average value of the correlation. $n_{k}$ is the number of the surrounding roads. $\bar{\mu }\left(d\right)$ is the average value of μ(k,d), and m=180 is the total number of target roads. The calculation results are shown in Figure 16.

Figure 16 shows that the results of the CCF-based calculation methods first exhibit a decrease with the increase in the time delay and then still maintain a strong correlation with a considerable time delay from the TR’s “specified time period” with a little change. The proposed DCF model shows that the correlation between the TR and the surrounding roads gradually decreases to 0 with an increase in the time delay.

However, due to the constant update of the traffic flow on the TR, the correlation between the traffic statuses of two different time and space regions (STRs) cannot last forever. Therefore, the result obtained by DCF could be more reasonable than CCF1 and CCF2.

### 5.5. Relationship between the Maximum Correlation and the Importance of Roads

Road network analysis based on complex theory has become an important method in the field of traffic flow research, especially in relation to spatial structure. In complex network theory, the more important the node (road) in the topology structure is, the greater the impact on other nodes (roads) [47]. Therefore, to verify the correlation reliably, the relationship between the correlation calculated by the three models and the importance of roads was analysed. The calculation process is as follows:

(1) The page rank algorithm (PRA) [48] and signal propagation algorithm (SPA) [49] were used to rank the surrounding roads according to the positions to the TR. The more important the road is, the higher it ranks with a smaller ID.
(2) Select the maximum correlation of the traffic state between each surrounding road and the TR with a finite number of time delays.
  (30) $$\psi_{k}\left(j\right)=\max\left(\begin{array}{ccccccc} r_{j,k}\left(0\right) & , & r_{j,k}\left(1\right) & , & \cdots & , & r_{j,k}\left(d_{\max}\right) \end{array}\right)$$
  where $\psi_{k}\left(j\right)$ is the maximum value of the correlation between TR k and road j with a time delay from 0 to $d_{\max}$,$d_{\max}=30$.

**Figure 17:**
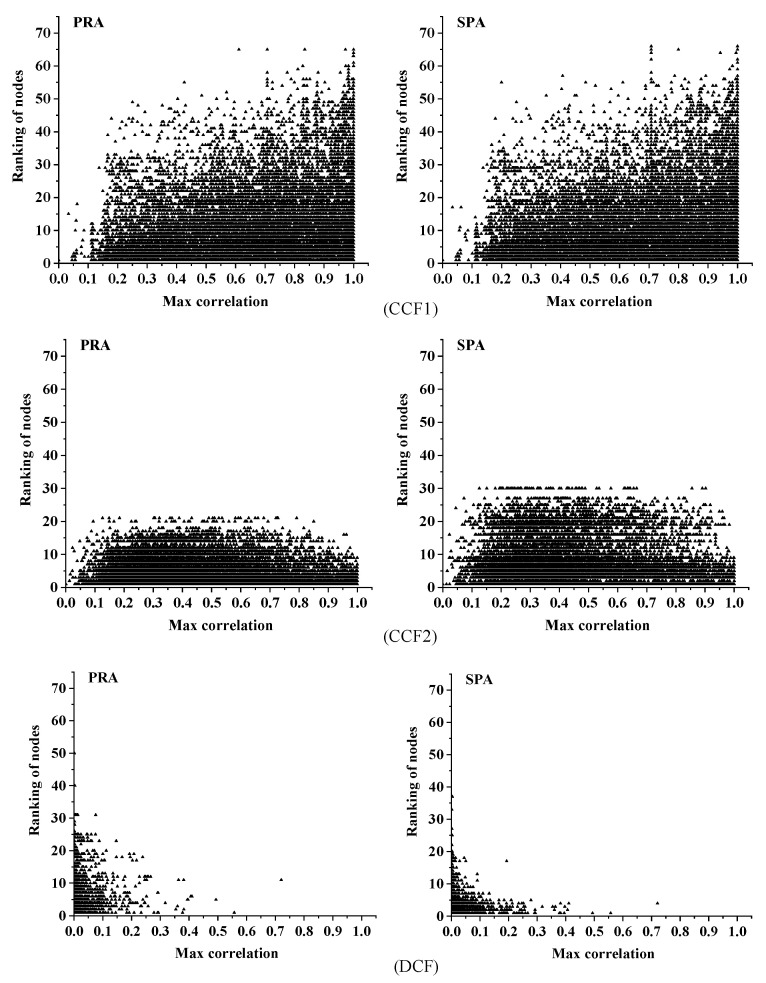
Relationship between the importance of nodes and their maximum relevance to the TR.

The relationship between the maximum correlation calculated by the three methods and the ranking of roads are shown in Figure 17.

In Figure 17, the maximum correlation calculated by the first CCF-based methods has no obvious relationship with the importance of roads. However, the DCF results show that the more important the road is, the more likely the maximum correlation will be.

According to complex network research [48,49], the more important a node is, the greater the impact it will bring when its state changes, and the closer its relationship with the surrounding nodes. Therefore, compared with the former two models, the results of the DCF proposed in this paper are more consistent with the understanding of node importance in complex network theory.

### 5.6. Relationship between the Correlation and Traffic Conditions

Urban traffic networks are complex network systems composed of physical road networks (static characteristics) and traffic demand networks (dynamic characteristics). The existing study has shown that in addition to the static physical structure of the road network, the dynamic operation of traffic flow can also reflect the degree of correlation between different roads [50]. To verify the models from the perspective of dynamic requirements, the relationship between the correlation and operation status of the TR was studied. A parameter named the non-vacant ratio was proposed, which is calculated as:

(31) $$\zeta_{i}\left(t\right)=\frac{1}{n}\sum_{\tau =t}^{t+n-1}\chi_{i}\left(\tau \right),\;\;\;\;\chi_{i}\left(\tau \right)=\left\{ \begin{array}{l} 1\;\;\;\;\;\;\;if\;\;\eta_{i}\left(\tau \right)\neq 1\\ 0\;\;\;\;\;\;\;if\;\;\eta_{i}\left(\tau \right)=1 \end{array}\right.$$

where $\zeta_{i}\left(t\right)$ is the non-vacant ratio between time t and t+n−1 of target road i. $\eta_{i}\left(\tau \right)$ is the state of the TR introduced in Section 3. This parameter reflects the degree to which the TR continuously participates in the information exchange of the road network.

**Figure 18:**
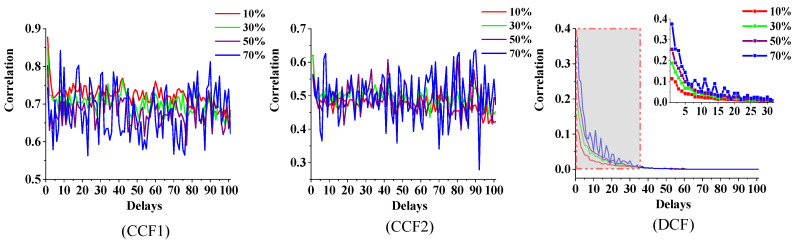
Relationship between the correlation and time delays for different non-vacant ratios.

The average value of the correlation between the TRs and their surrounding roads under each time delay condition was calculated. Then, the relationship between the average correlation and time delays with different non-vacant ratios of the TRs is shown in Figure 18. The abscissa and ordinate indicate the time delays and average correlation, respectively, and the dotted lines of different colours represent different non-vacant ratios.

The calculation results obtained by the two models, CCF1 and CCF2, show that the average correlation between the TR and its surrounding road network is not only irregular with the time delays but also unrelated to the non-vacant ratio. However, the DCF results show that the average correlation decreases with an increase in the time delay and increases with an increase in the non-vacant ratios.

According to the definition and related research, a larger non-vacant ratio in a certain period means that traffic flow exchange between the road and surrounding roads will be more frequent. Therefore, the TR is more likely to be affected by the traffic flow of the surrounding roads, which renders it more relevant to the surrounding road network. Thus, the DCF model proposed in this paper is more reasonable than the former two.

## 6. Conclusions

The calculation of the STC between roads affects not only the selection of the road networks but also the quality of data utilization of each road. Therefore, the provision of more reasonable correlation information for research, such as traffic speed prediction, is of significance. Traditional statistical-based calculation methods do not consider the actual operation regularity of the road network and dynamic characteristics of the traffic data. In addition, they seldom consider the characteristics of urban road network structure. Therefore, these studies have congenital deficiencies when explaining traffic relevance at the physical level.

Therefore, drawing support from the macro traffic model with the advantages of simple calculation and good physical interpretation, combined with the structural characteristics of urban road networks, this paper proposes an STC calculation method based on a traffic dynamic model in this paper for the first time.

Based on the traffic dynamic model (LWR model), the research explores the influence characteristics of urban traffic flow. The parameter of traffic complete influence time (TCIT) is proposed to measure the influence time between different roads for the first time. Another parameter of traffic correlation strength (TCS) was proposed based on the separation and confluence mechanism of intersections to effectively characterize the influence strength of different roads on the same road. Based on these two parameters, this paper creatively put forwards the STC calculation method according to whether the roads are directly adjacent. To integrate the structural characteristics of urban roads into the calculation process of STC, the calculation results are more explanatory.

Finally, the proposed method was verified against two common traditional methods through different experiments. The verification results show that the calculation method proposed in this paper has better interpretability for the STC between different roads and can better reveal the internal traffic operation characteristics of the road network.

This paper mainly focuses on the study of urban traffic correlation at the macro traffic level, but lacks the mechanism research on the correlation between non Lane level traffic within the road. The research of lane-level correlation can provide more detailed information for ITS, which is also the main content of future research for this paper. At the same time, combined with a more reasonable STC, traffic information prediction and path planning can also be further studied.

## Figures and Tables

**Table 1:** Flow ratio (%) of roads 2 and 1 to road 0.

Time	240	241	242	243	244	245	246	247	248	249	250	251	252	253	254	255	256	257	258	259
road 2	0	0	0	0	0	0	0	0	100	0	0	100	0	0	100	0	0	100	0	0
road 1	0	0	0	0	0	0	0	0	0	0	0	0	0	0	0	0	0	0	0	0

**Table 2:** Traffic correlation strength between road 2 and TR with different time delays.

Time Delays	$\xi_{2,0}\left(240\right)$	$\lambda_{2}$	$\gamma_{1}$	$\gamma_{2}$	$f_{2,0}$
0	249	0.1	1	1	0.1
1			0.9	1	0.09
2			0.8	0.5	0.04
3			0.7	0.5	0.035
4			0.6	0.5	0.03
5			0.5	0.33	0.0165
6			0.4	0.33	0.0132
7			0.3	0.33	0.0099
8			0.2	0.25	0.005
9			0.1	0	0
10			0	0	0

**Table 3:** Flow ratio (%) of roads 4 and 6 to road 2.

Time	240	241	242	243	244	245	246	247	248	249	250	251	252	253	254	255	256	257	258	259
road 4	0	0	0	0	0	0	0	0	100	0	0	100	0	100	100	0	100	100	0	0
road 6	0	0	0	0	0	0	0	0	0	0	0	0	0	0	0	0	0	0	0	0

**Table 4:** Flow ratio (%) of roads 1 and 2 to road 6.

Time	240	241	242	243	244	245	246	247	248	249	250	251	252	253	254	255	256	257	258	259
road 1	100	0	0	100	0	100	0	0	100	0	0	100	0	0	100	0	0	100	0	0
road 2	0	0	0	0	0	0	0	0	0	0	0	0	0	0	0	0	0	0	0	0
